# US drinking water quality: exposure risk profiles for seven legacy and emerging contaminants

**DOI:** 10.1038/s41370-023-00597-z

**Published:** 2023-09-22

**Authors:** Ronnie Levin, Cristina M. Villanueva, Daniel Beene, Angie L. Cradock, Carolina Donat-Vargas, Johnnye Lewis, Irene Martinez-Morata, Darya Minovi, Anne E. Nigra, Erik D. Olson, Laurel A. Schaider, Mary H. Ward, Nicole C. Deziel

**Affiliations:** 1 Harvard TH Chan School of Public Health, Boston, MA, USA.; 2 ISGlobal, Barcelona, Spain.; 3 CIBER epidemiología y salud pública (CIBERESP), Madrid, Spain.; 4 Universitat Pompeu Fabra (UPF), Barcelona, Spain.; 5 IMIM (Hospital del Mar Medical Research Institute), Barcelona, Spain.; 6 Community Environmental Health Program, College of Pharmacy, University of New Mexico Health Sciences Center, Albuquerque, NM, USA.; 7 University of New Mexico Department of Geography & Environmental Studies, Albuquerque, NM, USA.; 8 Department of Environmental Health Sciences, Columbia University Mailman School of Public Health, New York, NY, USA.; 9 Center for Science and Democracy, Union of Concerned Scientists, Washington, DC, USA.; 10 Natural Resources Defense Council, Washington, DC, USA.; 11 Silent Spring Institute, Newton, MA, USA.; 12 Occupational and Environmental Epidemiology Branch, Division of Cancer Epidemiology and Genetics, National Cancer Institute, National Institutes of Health, Rockville, MD, USA.; 13 Yale School of Public Health, New Haven, CT, USA.

**Keywords:** Drinking water, Exposure risk profiles, Social determinants, Water infrastructure, Water contaminants, Environmental disparities

## Abstract

**BACKGROUND:**

Advances in drinking water infrastructure and treatment throughout the 20^th^ and early 21^st^ century dramatically improved water reliability and quality in the United States (US) and other parts of the world. However, numerous chemical contaminants from a range of anthropogenic and natural sources continue to pose chronic health concerns, even in countries with established drinking water regulations, such as the US.

**OBJECTIVE/METHODS:**

In this review, we summarize exposure risk profiles and health effects for seven legacy and emerging drinking water contaminants or contaminant groups: arsenic, disinfection by-products, fracking-related substances, lead, nitrate, per- and polyfluorinated alkyl substances (PFAS) and uranium. We begin with an overview of US public water systems, and US and global drinking water regulation. We end with a summary of cross-cutting challenges that burden US drinking water systems: aging and deteriorated water infrastructure, vulnerabilities for children in school and childcare facilities, climate change, disparities in access to safe and reliable drinking water, uneven enforcement of drinking water standards, inadequate health assessments, large numbers of chemicals within a class, a preponderance of small water systems, and issues facing US Indigenous communities.

**RESULTS:**

Research and data on US drinking water contamination show that exposure profiles, health risks, and water quality reliability issues vary widely across populations, geographically and by contaminant. Factors include water source, local and regional features, aging water infrastructure, industrial or commercial activities, and social determinants. Understanding the risk profiles of different drinking water contaminants is necessary for anticipating local and general problems, ascertaining the state of drinking water resources, and developing mitigation strategies.

**IMPACT STATEMENT:**

Drinking water contamination is widespread, even in the US. Exposure risk profiles vary by contaminant. Understanding the risk profiles of different drinking water contaminants is necessary for anticipating local and general public health problems, ascertaining the state of drinking water resources, and developing mitigation strategies.

## INTRODUCTION

In 2010, the United Nations General Assembly explicitly recognized the human right to affordable and safe drinking water. The 20th and early 21st centuries saw major advances in the provision of reliable water to the developed and developing world. Achieving universal access to basic drinking water remains a critical global health goal. As access to drinking water becomes more widespread, concerns about chronic health issues from chemical contamination of drinking water provided through modern water systems become more paramount. Understanding the risk profiles of different drinking chemical contaminants is a necessary basis for assessing the state of drinking water resources.

In this narrative review, we begin with an overview of the configuration and governance of US public drinking water systems. For comparison, we briefly summarize the World Health Organization’s (WHO’s) approach to drinking water guidelines. To illustrate the variety of risks that may occur, we describe the exposure risk profiles of seven commonly-occurring chemical contaminants each affecting millions of Americans: arsenic, disinfection by-products, fracking-related substances, lead, nitrate, PFAS and uranium. We selected these contaminants to represent legacy (i.e., well-known contaminants that are generally regulated) and emerging chemicals (i.e., contaminants more recently identified as environmental health threats and often lacking regulations), threats to ground and surface water, issues of local and regional contamination, natural and anthropogenic sources, organic and inorganics, and those related treatment and distribution features or social determinants (that is, the non-medical factors that influence health outcomes such as income, race or ethnicity, housing and employment and the wider set of forces and systems shaping the conditions of daily life). We conclude with a discussion of cross-cutting issues and challenges: aging and deteriorating water infrastructure; problems related to children, schools, and childcare settings; climate change; disparities in access to clean, reliable, safe drinking water; inconsistent regulatory enforcement; inadequate or outdated health assessments; preponderance of small systems; large numbers of substances within chemical groups; uneven enforcement of US drinking water standards; and disenfranchisement of Indigenous communities.

## US PUBLIC DRINKING WATER SYSTEMS

In the US, there are about 150,000 public water systems (PWSs), i.e., those that serve at least 15 service connections or an average of at least 25 people for at least 60 days a year (Table 1). They may be owned publicly, by a governmental or semi-governmental entity, or privately. A community water system (CWS) serves the same population over the course of the year, while a noncommunity system, such as a restaurant or campground, serves different populations. CWSs account for one-third of PWSs (~49,600 of 150,000) but serve about 320 million Americans, approximately 95% of the US population.

CWSs are not evenly distributed across service-size populations (Table 1). Most CWSs (91%) are small-medium, serving under 10,000 people each, but together only serve 16% of the US population (52 million people), while the largest 9% of CWSs provide water to 83% of the US population (267 million people) [1]. Water source is not evenly divided between surface and groundwater systems; while 77% of CWSs are supplied by groundwater, they only serve 28% of the population (Table 2). In addition to the 320 million Americans served by PWSs for at least some of their water, >43 million people (~15% of the US population) rely on domestic (private) wells for residential drinking water [2]. This review focuses primarily on CWSs, including small and very small systems, but the large number of non-community systems and private wells poses additional and unique challenges. An added complexity with regard to exposure and health assessments is that many people are served by multiple water systems at home, work, school, and other locations; consequently, the total population served by PWSs in the US exceeds the estimated population.

Under the US Safe Drinking Water Act (SDWA) [3], the US Environmental Protection Agency (EPA) sets legal limits for contaminants in public drinking water known as the Maximum Contaminant Level (MCL). For each regulated contaminant, EPA sets a health-based Maximum Contaminant Level Goal (MCLG), the level at which an adult can regularly consume drinking water over a lifetime with an adequate margin of safety. The MCL reflects the level closest to the MCLG that CWSs can feasibly achieve using the best available technology; the 1996 Amendments to the SDWA allow EPA to use cost-benefit analysis to set an MCL that is less stringent than is feasible. If EPA decides setting a numerical MCL for a contaminant is infeasible, EPA can issue a Treatment Technique instead. EPA has promulgated standards for about 100 contaminants in drinking water (Table 3) [4]. This is a small fraction of the approximately 700 identified disinfection by-products [5], 1200 chemicals reportedly used or produced by fracking [6], 14,700 PFAS [7], and other chemicals in commercial use. While not all of these compounds are likely to be present in drinking water, this suggests that EPA’s current regulatory structure may be missing many chemical contaminants of concern. EPA uses the Unregulated Contaminant Monitoring Program to collect occurrence data for up to 30 contaminants suspected to be present in drinking water, but that do not have health-based standards. Every five years, EPA reviews the contaminant list to determine if any should be considered for regulation.

## GLOBAL DRINKING WATER STANDARDS

The WHO does not recommend a uniform international enforceable standard for drinking water. Instead, WHO advocates a local risk-benefit approach (qualitative or quantitative) for establishing national standards and regulations according to local needs and resources [8]. WHO therefore issues guidance for developing national and regional drinking water standards, recommends periodic review of these standards, and suggests that updates can be made readily. While WHO has issued guidelines for numerous drinking water contaminants, none are legal or enforceable standards, as WHO is not a regulatory body. However, many countries rely on WHO guidelines as the basis for their drinking water standards [8]. Countries and territories that specify their own parameter values for drinking-water quality do so in a variety of formats: regulations, standards, specifications, laws, decrees, requirements, and norms. With very few exceptions, the majority set regulatory values equal to or more stringent than the WHO Guideline [9, 10].

## CONTAMINANT PROFILES

### Arsenic

#### Sources and health effects.

Inorganic arsenic is a known human carcinogen causally associated with cancers of the skin, bladder, and lungs, and epidemiologic evidence supports a potential association with cancers of the kidney, breast, pancreas, and liver [11, 12]. Chronic exposure is also associated with respiratory disease, cardiovascular disease, adverse birth outcomes, metabolic disorders and diabetes, impaired immunological functioning, kidney disease, and adverse neurocognitive outcomes [13, 14]. Risk of some outcomes including cancer likely persist for chronic exposure to water arsenic at concentrations at or below EPA’s MCL of 10 μg/L [15, 16].

#### Exposure risk profile.

The significant spatial variability in water arsenic concentrations across the US reflects variability in geologic, biogeochemical, hydrologic, and climatic conditions that influence geogenic arsenic prevalence in bedrock and solubility, (e.g., arid oxidizing environments in the Southwestern US, and humid reducing environments and alkaline pH in the Northeast) [17]. Arsenic is detectable in over 50% of CWSs contributing to the EPA’s Six Year Review database (>36,000 CWSs) [18]. Approximately 2.6% of CWSs report arsenic concentrations exceeding the MCL (10 μg/L). An estimated 85% of PWSs rely on groundwater sources. Predicted arsenic levels in domestic wells are strongly associated with CWS concentrations using the same groundwater resources [19]. Hardrock mining processes and mine waste also contribute to surface and groundwater arsenic in the Southwest and Great Plains regions of the US, especially near Indigenous communities [20].

Significant sociodemographic and regional inequalities in CWS arsenic exposures have been identified across the US. In 2009–2011, mean arsenic concentrations were 1.70 μg/L nationwide and were highest in: systems serving communities categorized as *Semi-Urban, Hispanic* (3.40 μg/L); communities in the Southwestern US (3.18 μg/L); communities with less than 500 residents; communities reliant on groundwater sources; and incarcerated populations in the Southwest (2006–2011, mean 6.41 μg/L) [18]. At the county level, a higher proportion of Indigenous or Hispanic/Latino residents was associated with higher CWS arsenic concentrations, after adjustment for socioeconomic vulnerability [21]. A California study similarly found that higher proportions of Latino residents were associated with significantly higher CWS arsenic concentrations [22]. Similarly, a higher proportion of non-Hispanic Black residents was also associated with higher CWS arsenic concentrations in the Southwestern US, while concentrations were inversely associated with higher proportions of non-Hispanic White residents both nationwide and regionally. Higher county-level high school diploma attainment was associated with lower CWS arsenic concentrations; this association was modified by region, likely reflecting regional/local differences in socioeconomic context and drinking water infrastructure [23].

#### US regulatory context.

Evidence from EPA violation records, routine compliance monitoring records, and urinary biomarkers of water arsenic exposure indicates that public water arsenic exposure declined significantly following the reduction of EPA’s MCL from 50 to 10 μg/L (effective 2006), consistent with the WHO guideline of 10 μg/L [18, 24–26]. The largest exposure reductions occurred for Mexican-American residents (36% reduction, compared to 17% overall) and for CWSs with the highest baseline arsenic concentrations, including those serving New England (mean 37% reduction) and Alaska and Hawaii (mean 24% reduction).

EPA’s MCLG of 0 μg/L for arsenic reflects that there is no safe level of exposure to carcinogens, and mounting epidemiologic evidence supports that the current MCL is inadequate to protect human health. Denmark and the states of New Jersey and New Hampshire set more health protective MCLs of 5 μg/L, and water utilities in the Netherlands voluntarily adopted 1 μg/L. In setting the 5 μg/L MCL, the New Jersey Department of Environmental Protection (NJDEP) cited the National Academy of Sciences’ 2001 report that a water arsenic concentration of 0.003 μg/L is estimated to result in a one-in-one-million excess lifetime risk of lung and bladder cancer [27]. NJDEP considered available testing and treatment technologies but not cost-benefit analysis [22]. Significant uncertainties and a lack of overall scientific consensus remain regarding the risk assessment for inorganic arsenic. The current EPA Integrated Risk Information System (IRIS) cancer slope factor for arsenic (1.5 per mg/kg bodyweight-day) relates to skin cancer only, while the 2010 proposed slope factor (25.7 per mg/kg bodyweight-day) corresponds to combined lung and bladder cancers and reflects increased susceptibility for women; synergistic effects for tobacco smoking are not considered in either [28].

#### Unique and shared challenges.

Climate change poses significant challenges to reducing water arsenic concentrations, especially in the Southwest where water arsenic levels are already high. Wildfires and extended drought conditions caused by climate change are likely to concentrate arsenic and other inorganic contaminants as water levels decrease in groundwater [19, 29]. Additional epidemiologic studies of drinking water arsenic at low- to moderate levels relevant for US populations, especially investigating cancer and cardiovascular disease, in diverse US populations would further inform risk assessment efforts.

### Disinfection by-products

#### Sources and health effects.

Disinfection of drinking water is necessary to prevent waterborne infections. However, disinfectants are highly reactive and interact with organic matter, bromide, nitrogenous compounds and other precursors to form unintended disinfection by-products (DBPs) [30, 31]. DBP concentrations can vary substantially based on source water, disinfectant and treatment practice. Chlorine is a cost-effective disinfectant widely used worldwide, and trihalomethanes (THMs) followed by haloacetic acids (HAAs) are the most prevalent chlorination by-products. THMs comprise chloroform, dibromo-chloromethane, bromodichloromethane, and bromoform [32]. Chloroform is reported to be the dominant THM (up to 90%) in many areas worldwide, while brominated THMs are the more abundant species elsewhere, such as in Middle East countries, associated with high concentrations of bromide ions in raw water [33]. Local bromide discharges from industrial sources, including coal-fired power plants, oil and gas extraction activities, and textile mills, will impact DBP risks [34].

Alternative disinfectants may decrease THM formation but may encourage formation of other DBPs, such as chlorate and chlorite (from chlorine dioxide), nitrogenous DBPs (chloramines), bromate (ozone), iodinated and brominated aromatic DBP (chlorine dioxide). Chloramines are widely used in the US as an alternative to chlorine to reduce THM formation. Some of these DBPs show higher toxicity at lower concentrations than THMs and HAAs [33]. Among the ~700 identified DBPs, only 4 THMs, 5 HAAs, bromate, chlorate, and chlorite are currently regulated in the US and/or the European Union (EU) (Table 3) [35, 36].

Long-term exposure to THMs, as a marker of DBP exposure, has been consistently associated with bladder cancer risk in epidemiological studies [37]. DBP exposure has also been linked to pregnancy and reproductive outcomes, but evidence is mixed [38, 39]. The WHO International Agency for Research on Cancer has classified chloroform and bromodichloromethane as possible human carcinogens [40]. Iodinated DBPs are more toxic in mammalian cells than their chlorinated and brominated analogues [41]. Iodinated DBPs may be among the most genotoxic and cytotoxic DBPs, with iodoacetic acids potentially the most genotoxic of all DBPs [42]. Endocrine disruption and adverse reproductive and developmental impacts are also seen [43–45]. Some nitrogenous DBPs, including haloacetonitriles, haloaceta-mides, and halonitromethanes are more toxic and carcinogenic than THMs and HAA [43, 46]. In particular, the toxicity of haloacetamides is estimated to be 142 times higher than HAAs [47].

#### Exposure risk profile.

DBP formation generally is higher in surface than groundwater systems due to higher levels of natural organic matter. The composition of organic matter and other water constituents in raw water affects which DBPs are formed. In particular, hydrophobic organic matter (e.g., high molecular weight organic materials) has higher potential to form THMs, nitrogenous DBPs and aromatic DBPs than hydrophilic organic matter. Occurrence of bromide and iodide ions promotes the formation of brominated and iodinated DBPs, respectively, while ammonia in water favors nitrogenous DBP formation [46].

Seasonal fluctuations (involving variations of surface water quality and water temperature) influence DBP formation. DBPs tend to show higher concentrations in summer than winter and certain DBP groups exhibit distinct seasonal patterns. Hydrophobic natural organic matter is positively correlated with air temperature whereas the hydrophilic natural organic matter shows a reverse trend [48]. Temperature increases the reaction rate between organic matter and chlorine, increasing THM formation. HAA formation, however, increases with temperature up to 20 °C then decreases. High temperatures also increase THM volatilization. Likewise, high pH enhances THM formation as well as the hydrolysis of other DBPs into THMs and HAAs, while low pH increases HAA formation [33, 49, 50].

THM and HAA formation increases with disinfectant dose and residence time in the distribution system, so distal parts of the distribution system generally have higher DBP levels. DBP exceedances occur more often in small systems, which have fewer resources for treatment to reduce DBP levels, such as filtering organic matter prior to disinfection [51]. DBP formation in the distribution system may be higher with polyethylene pipes [52] and with increasing diameter and pipe age [53].

Tap water is also used for showering, bathing, dishwashing and cooking. While exposure to non-volatile DBPs occurs predominantly through ingestion, exposure to volatile and skin-permeable DBPs (e.g., THMs) also occurs through dermal absorption and inhalation [32, 54, 55]. Exposure to THMs in swimming pools has been evaluated (measuring THMs in blood, exhaled air and urine), but the relative importance of different exposure routes remains inconclusive [56].

#### Unique and shared challenges.

The pervasive presence of DBPs in drinking water poses significant concerns for human health. Even regulated DBPs lack comprehensive toxicological evidence, and while data indicate that some unregulated DBPs may pose greater risks that those currently regulated, the majority of emerging DBPs remain poorly understood. Furthermore, the potential interactions among the 700 identified DBPs in drinking water, have not been adequately examined individually or in mixtures. Synergistic effects of climate change (e.g., increasing temperature, more frequent and severe flooding and droughts), acidification of soil and surface water, land use change and other anthropogenic pressures all impact water quality and consequently, water treatment and DBP formation [57]. Precipitation and temperature are the main climate factors affecting water quality. The increase in atmospheric temperature and warming of surface waters is linked to eutrophication and increased microbial activity and dissolved organic carbon. Drought has been shown to significantly impact water chemistry, including higher levels of hydrophilic organic matter [58]. Ultimately, all these factors impact the concentration and composition of organic matter and consequently, DBP formation [57]. Aging and outdated water infrastructure and the large number of small systems are also challenges.

### Fracking-related substances (Unconventional oil and gas development)

#### Sources and health effects.

Unconventional oil and gas development (UOGD), commonly called “fracking”, is a method for extracting oil and natural gas from deep, low permeable geologic formations, requiring more intense stimulation compared to more accessible, conventional reservoirs [59]. In the US, there are approximately 150,000 active UOG wells, and more than 9 million people rely on drinking-water sources located within 1.6 km (1 mile) of a UOG well [59]. Water contamination from UOGD remains a major community concern [60].

Fracturing fluids and wastewater used or produced by UOGD may contain toxic, radioactive, endocrine-disrupting, and/or carcinogenic chemicals [6, 61]. Potential water contamination events include surface spills of fracturing fluids or wastewater at the well site, release of improperly treated wastewater, and leaks in well infrastructure [62]. An estimated 1–4% of UOG wells have reported spills [63, 64] and, based on Pennsylvania data, approximately 20% have a non-administrative violation [65]. These are uncommon events, and multiple groundwater monitoring studies conducted in regions with UOGD have not found evidence of widespread contamination [66–69]. However, specific instances of groundwater and surface water impairments have been identified [70–72].

#### Exposure risk profile.

Numerous epidemiologic studies have observed an increased risk of adverse health effects including adverse perinatal outcomes, childhood cancer incidence, hospitalizations, asthma exacerbations, mental health issues, and mortality in the elderly in relation to proximity to UOGD sites [59]. The extent to which these associations may be related to water is unclear, because most studies have used aggregate proximity-based metrics to assign exposures, which are not specific to any hazard [59]. A few epidemiologic studies have focused specifically on the drinking water exposure pathway. Two studies of pediatric health outcomes applied a novel water-pathway specific metric that restricts the analysis to UOGD wells that are hydrologically connected to the watershed of a residence [66, 73]. This exposure metric is most relevant for groundwater. Another study found that proximity of community drinking water sources to UOGD wells was associated with greater likelihood of a variety of adverse birth outcomes [74, 75]. More research is needed to understand whether the increased health risks observed in populations living in the vicinity of UOGD are attributable to drinking-water exposures, other hazards, or a combination of factors.

#### Unique and shared challenges.

In the US, UOGD often occurs in rural areas where homes are served by private (domestic) drinking water wells, which are not subject to federal regulations and monitoring [76], or by small water systems. Therefore, available data are quite limited, and the data that are available tend to focus on the few chemicals that are regulated, which are only a tiny subset of the approximately 1200 chemicals reportedly used or produced by UOGD.

Although chemical disclosure policies have improved, researchers still lack a complete and consistent inventory of chemicals used in UOGD [77]. Furthermore, contaminants also include transformation products and naturally occurring chemicals mobilized during the development process [78]. Some researchers have mined existing voluntary reporting databases and created their own datasets [79].

Proximity to UOGD wells, often emphasized for sampling and in risk estimation, may be an inadequate surrogate for predicting exposure given geologic heterogeneity and topographical variations producing the complex flow paths and stochastic nature of contamination events [66]. Applying physically based hydrological models demonstrates that unlike the typical circular buffers used in exposure and epidemiologic studies, the groundwater capture zones exhibit more of a surfboard shape [80]; better modeling the capture zone could improve identification of homes more vulnerable to contamination and inform sampling locations. In addition, application of machine learning techniques to available monitoring data can help identify hotspots [81, 82].

Finally, information on the locations and descriptions of where violations and spills occur is not available in real time and therefore timely sampling in response to a potential contamination event is not feasible. In addition, violations data are not available in a consistent format across states, posing another challenge to multi-state research. Further, chemicals disperse at varying rates (particularly in groundwater), so a single collected sample may be unlikely to coincide with release of a plume of multiple contaminants, potentially necessitating repeated measures.

### Lead

#### Sources and health effects.

Lead is a widely used element with thousands of applications. So closely associated with the conveyance of water, the very word ‘plumbing’ derives from its Latin name, plumbum. Lead is also highly toxic and associated with adverse health endpoints across virtually all body systems, including nervous, cardiovascular, renal, immunological, hematological and reproductive/developmental systems in men and women, in adults and children [83–85]. Lead has been classified as a probable human carcinogen by EPA since 1988 based on rodent toxicology data [83].

#### Exposure risk profile.

Lead is usually a corrosion by-product in drinking water related to water’s natural corrosivity and lead’s extensive use in plumbing components such as pipes, solder, brass and bronze [86], faucets [87, 88], galvanized steel pipe coatings [89, 90], valves and meters. Lead has been progressively restricted from plumbing use in the US during the past few decades, but its durability means that an estimated 9.2 million lead pipes installed in the late 1800’s to early 1900’s remain servicing US homes [91, 92].And until banned in 1986 [93], lead solder joining copper pipes was practically ubiquitous in the US.

Studies evaluating multiple lead exposure sources from within the house found that, when present, lead pipes contribute the most lead to drinking water [94]. Lead pipes carrying water from the water main to the residence (lead service lines) and lead pipes inside homes were often installed in cities that expanded greatly during the Industrial Revolution [95] therefore home age and the history of the urban area are factors to consider. However, lead pipes continued to be installed in the US until banned in 1986, including locales such as Chicago that required lead service line installation until then. Beginning in the 1930s, copper pipes replaced lead pipes as the most common residential piping material; lead solder was used to join them. The combination of copper and lead produces galvanic corrosion that is associated with high lead leaching potential and elevated water lead levels (WLLs).

All water is corrosive, but the degree of corrosivity varies. Principal factors include pH, alkalinity and hardness of the water [96]. Seasonality is evident in WLLs [97, 98]. The warmer the temperature the greater the potential for lead leaching; consumption of drinking water also increases in the summer.

#### US regulatory context.

EPA issued the Lead and Copper Rule (LCR) in 1991. Lead contamination of tap water relates mostly to water corrosivity and corrosion within the lead service line and residence; and water lead levels vary due to stagnation time, temperature, extent of lead plumbing components, and other factors. Citing some of these factors, EPA decided not to set an MCL for lead. EPA’s Lead and Copper Rule (LCR), instead, established a Treatment Technique requiring systems to control the corrosiveness of their water. If more than 10% of the tap water samples exceed the Action Level (AL) of 0.015 mg/L, water systems must take additional steps. The AL was based upon feasibility; it is not enforceable and exceeding it is not a violation of the SDWA. In January 2021, EPA revised the LCR [99] and almost immediately agreed to review it to address shortcomings [100]. The MCLG is zero based upon both lead’s carcinogenicity and that no safe level of exposure has been determined.

The SDWA only regulates PWSs. Homes with private wells, which are not covered under the SDWA, are less likely to have lead pipes than older urban areas. On the other hand, they may be more vulnerable to use of leaded solder and are less likely to use a corrosion inhibitor even with very acidic water; they may also have lead in their water pumps [101]. One study found that WLLs from private wells may be higher than those in adjacent public water systems [102].

Numerous studies show the clinical significance of exposures to even low WLLs across a range of effects. WLLs are associated with cognitive performance in children [103], renal function in dialysis patients [104], adverse birth outcomes [105], iron deficiency in patients with End Stage Kidney Disease [106], and the likelihood of juvenile delinquency [107]. Water-lead outbreaks are attested to raise general population BLLs [108, 109] and BLLs are also associated with WLLs in non-crisis circumstances [110]. Violations of the LCR are common. The Government Accountability Office (GAO) reported that at least 10% of the water systems subject to the LCR had at least one open violation of the rule [111]. This is likely an underestimate as GAO has repeatedly found that EPA’s enforcement data are incomplete, especially related to compliance with the LCR [111–113]. This is consistent with EPA’s finding that only 8% of LCR treatment technique violations reported to states are passed on to EPA. Similarly, a 2016 report found that over 5300 CWSs serving an estimated 18 million Americans violated the LCR that year [114]. A later study found that 186 million Americans receive water from CWSs with detectable lead contamination [115]. A familiar pattern is evident in violations of the LCR in the US: minoritized and low-income communities bear an increased risk of receiving poorer quality drinking water and of having lead pipes [116–118].

#### Unique and shared challenges.

Climate change effects include the acidification of the natural world, reducing the pH in air, water and soil. Lower pH is associated with increased lead mobility and bioavailability [119–121]. Hence, climate change and global warming may increase water lead levels both through thermal and biochemical mechanisms as well as increasing lead’s mobility and bioavailability. Global warming will likely increase water consumption, also.

Disparities exist in lead exposure, enforcement issues and problems with school drinking water. US lead exposures from drinking water appear to be widely underestimated related to systematic poor monitoring, reporting and enforcement [122]. Less data are available on lead contamination of drinking water outside the US, but there is evidence of underestimation of lead contamination in EU drinking water, also [123]. The WHO provisional guideline value is 10 μg/l, set in 2011; sampling protocols differ between the US and WHO [124]. Until the remaining 9.2 million lead pipes are replaced and effective corrosion control is widely adopted, drinking water will remain a significant lead exposure source.

### Nitrate in drinking water

#### Sources.

Nitrate levels in water resources have increased world-wide from applications of inorganic fertilizer and animal manure in agricultural areas [125]. Contamination sources also include septic systems that do not effectively remove nitrogen and discharges from wastewater treatment plants [126], as well as atmospheric deposition of nitrogen oxides and fertilizer use on lawns, golf courses, and parks.

#### Exposure risk profile.

Private wells typically have higher nitrate concentrations than CWSs due to their shallower depth. High nitrate concentrations (near/exceeding the MCL) are most common in shallow (<100 feet) private wells located in agricultural areas because of nearby nitrogen sources (fertilizer use, animal operations, septic systems) [127]. Treatment of private wells is the responsibility of the property owner, leading to racial/ethnic, rural/urban, and socioeconomic disparities in access to safe drinking water. Some states provide resources, such as subsidized water test kits, to private well users. However, state-level regulation of private wells varies dramatically, and private well users are often unaware of the resources available to them [128].

A 2019 study [129] evaluating nitrate exposures in US CWSs estimated that about 5.6 million people were exposed to water with ≥5 mg nitrate-nitrogen (NO3-N)/L (more than half the MCL of 10 mg NO3-N/L) between 2010 and 2014. Hispanic/Latino residents were more likely to be served by CWSs with elevated nitrate levels. Disproportionately high exposure to nitrate-contaminated water among Hispanic/Latino communities has also been identified in the Yakima Valley of Washington State and San Joaquin Valley of California, among other areas [130]. Additional research has documented cases of poor water quality, including high nitrate concentrations, in drinking water used by migrant worker communities, Alaska Native villages and other Tribal lands, and in colonias along the US-Mexico border [130]. Limited measurement data characterizing residents using private wells presents challenges but recent advances in exposure modeling have proved useful for identifying exposure disparities [131–133].

#### US regulatory context and health concerns.

The US recommended standard for nitrate in drinking water was originally set in 1962 by the US Public Health Service as 10 mg NO3-N/L, based on infant methemoglobinemia. EPA’s subsequent MCL only considered this outcome and was based on a no-adverse-effect concentration for drinking water used to prepare formula for infants <6 months of age with no margin of safety; other health effects were not considered [134, 135]. The MCL has not been revised since it was promulgated in 1975 [135]. The literature investigating the health effects of nitrate exposure has expanded greatly since the MCL was set [136]. Nitrate ingested from drinking water may increase the risk of birth defects and some cancers because nitrate is a precursor in the formation of N-nitroso compounds (NOC), many of which are teratogens and carcinogens. NOC are formed in the body (a process called endogenous nitrosation) when nitrate is consumed in the absence of antioxidants that inhibit their formation [137]. Among epidemiologic studies with individual-level data, seven studies (in Australia, Canada, California, Texas, and Denmark) found increased central nervous system (CNS) malformations in children whose mothers consumed drinking water with high nitrate concentrations during pregnancy. In six studies, the increase in CNS malformations occurred at levels below the MCL [136, 138–140]. Studies of spontaneous abortion, fetal growth, and birth weight have been more limited and had mixed results [136, 139, 140].

In 2006, the International Agency for Research on Cancer (IARC) concluded that when ingested under conditions that result in endogenous nitrosation, nitrate and nitrite are probably carcinogenic to humans [137]. Since the IARC review, there have been more than 20 studies of incident cancer mostly in the US and Europe. Colorectal cancer is the most well-studied, with four of five studies finding increased risks [136, 141]. Studies of other incident cancers were fewer; however, positive associations at levels below the MCL were observed for cancers of the bladder [136, 142], kidney [136], childhood and adolescent/young adult brain [143–145], ovary and thyroid [136].

Unlike nitrate in drinking water, nitrate naturally present in food (mostly in fruits and vegetables) is consumed together with antioxidants, vitamins and polyphenols that inhibit endogenous nitrosation [146]. In controlled longitudinal feeding studies, high nitrate intake through consumption of high-nitrate vegetables such as beets and dietary supplements has been shown to reduce hypertension and may play a role in the protective effect of vegetables on cardiovascular disease risk [147, 148]. However, hypertension and nitrate ingestion through drinking water sources has not been studied. Clinical and subclinical hypothyroidism have been linked to higher nitrate intake from drinking water in several studies [136] and deserve further study.

Additional studies of cancers, thyroid disease, and birth outcomes/defects that have shown the most consistent associations with drinking water nitrate are needed to further elucidate risks below the MCL. Evaluating subgroups with higher endogenous nitrosation will improve inference. Methods for quantifying the nitrate-reducing bacteria in the oral microbiome and characterizing genetic variation in N-nitroso compound metabolism hold promise for identifying these high-risk groups in epidemiologic studies.

#### Unique and shared challenges.

Over the past several decades, nitrate levels in many ground and surface waters increased despite efforts to reduce nitrogen inputs [127]. Nitrate concentrations are expected to increase in aquifers used for drinking water as the contamination in shallow groundwater moves to deeper aquifers [127]. Disparities in exposure, an outdated health assessment, and large numbers of contaminated small systems and private wells are among the shared challenges related to nitrate. Efforts to understand the disproportionate impacts of nitrate exposure will help inform future policy and regulations to limit sources of nitrate contamination, decrease exposure, and alleviate public health harms.

### PFAS (per- and polyfluoroalkyl substances)

#### Sources and health effects.

Per- and polyfluoroalkyl substances (PFAS) are a major class of contaminants of concern in drinking water across the US and globally. PFAS as a class are generally considered “emerging” although some individual chemicals have state-level MCLs or have been phased out of commercial production may be classified as “legacy” pollutants. PFAS exposures are widespread; according to the National Health and Nutrition Examination Survey (NHANES), over 98% of the US population has detectable levels of PFAS in their blood [149]. In areas with contaminated water supplies, drinking water is a major contributor to PFAS exposure, although exposures can also come from diet, consumer products, and building materials. Dubbed “forever chemicals” because of their extreme persistence in the environment, PFAS also raise concerns due to far-reaching toxicity and bioaccumulation potential, with certain long-chain PFAS having human half-lives in blood on the order of years [150]. Exposures to some PFAS have been associated with adverse health outcomes across many major systems in the body, including immunotoxicity, dyslipidemia, changes in thyroid hormone levels, decreased birth weight, and testicular and kidney cancer [151–153]. Much of the available toxicological and epidemiological evidence is based on a relatively small number of long-chain PFAS (often those considered “legacy” PFAS), especially perfluorooctane sulfonate (PFOS) and perfluorooctanoic acid (PFOA), which have been phased out of production in the US and Europe, but reportedly are still being produced internationally and imported into the US in consumer goods [154]. However, a growing body of evidence raises concerns about the toxicity of “emerging” alternative PFAS being used as replacements [155, 156].

#### Exposure risk profile.

The highest levels of PFAS in drinking water have been found close to industrial facilities where PFAS are manufactured or processed and sites with discharges of aqueous film forming foam (AFFF) at military bases, major airports, and other fire training areas [157]. PFAS are widely used in consumer items, such as stain-resistant carpets and upholstery, food packaging, apparel, and cosmetics. An increasing number of PFAS contamination sites have been linked to waste disposal, including land-applied biosolids, effluent from wastewater treatment plants and septic systems, and landfill leachate [158].

PFAS have increasingly been detected in PWSs as analytical sensitivity has improved and testing has become more wide-spread. In the third round of EPA’s Unregulated Contaminant Monitoring Rule (UCMR3) in 2013–2015, which included six (mainly legacy) PFAS compounds, only 4% of PWSs reported detections above minimum reporting limits (MRLs) [157, 159]. However, this testing greatly underestimated the extent of PFAS in PWSs as the MRLs were relatively high (10–90 ng/L) and most smaller PWSs (≤10,000 customers) were not included. A more recent analysis estimated that 18–80 million Americans are served by PWSs delivering ≥10 ng/L of PFOS and PFOA and that the water of 200 million Americans has concentrations of PFOS and PFOA ≥ 1 ng/L [160, 161]. In 2023, EPA estimated that from 70 to 94 million people in the US are exposed to six PFAS of concern in their drinking water at elevated levels [162]. Private wells also can be vulnerable to PFAS contamination, even in areas without industrial activity [126] but little testing has been conducted on private wells [163]. PFAS contamination of drinking water supplies is emerging as an environmental justice concern. A recent analysis of monitoring data from 7873 CWSs in 18 US states found that detection of several PFAS is positively associated with the number of PFAS sources and proportions of people of color (Hispanic/Latino, non-Hispanic Black) who are served by these water systems. There are also disparities in the extent of PFAS testing; a smaller proportion of Tribal PWSs were included in the UCMR 3 testing compared to non-Tribal PWSs, and this difference will likely persist in the UCMR 5 testing currently underway [164].

Understanding the characteristics of PWSs most likely to have PFAS can help prioritize PFAS testing in areas where drinking water is most vulnerable. In the UCMR 3 testing, detection frequencies were twice as high among PWSs that relied on groundwater sources compared to surface water, although this testing found that short-chain PFAS were more frequently found in surface water systems [157]. These patterns vary in different regions of the world; nationwide testing of Swedish drinking water found that detection frequencies in surface water systems were twice as high as for groundwater systems [165]. In a 2022 study of groundwater in the eastern US, PFAS were detected in 60% of public supply wells and 20% of private wells, and PFAS detections were correlated with nearby urban land use, tritium (a marker of recent recharge), volatile organic compounds, and pharmaceuticals [166].

#### US regulatory context.

In the absence of enforceable federal drinking water standards, a regulatory patchwork emerged as some individual states adopted their own regulations. In 2016, EPA lowered its non-enforceable health advisories for PFOA and PFOS from 400 ng/L and 200 ng/L, respectively, to 70 ng/L for the two compounds individually or combined. Between 2016 and 2022, 18 US states adopted health advisories or enforceable standards at levels lower than 70 ng/L for PFOA and PFOS and/or for other PFAS compounds, mainly in the range of 10–20 ng/L for several PFAS compounds, individually or combined [167]. In 2022, EPA issued drastically stricter interim health advisories of 0.004 ng/L for PFOA and 0.02 ng/L for PFOS, noting “the levels at which negative health outcomes could occur are much lower than previously understood,” and finalized health advisories for two other PFAS [168]. In March 2023, EPA issued long-awaited draft MCLs of 4 ng/L for both PFOA and PFOS (individually), reflecting the “lowest feasible quantitation level.” [162] At the same time, EPA also issued a third MCL for four additional PFAS (PFHxS, PFNA, PFBS, and GenX chemicals) using a Hazard Index value of 1. The Hazard Index approach is routinely applied in risk assessment settings, but this represents the first proposed use for setting drinking water MCLs and represents a step towards more of a class-based approach by moving beyond a one-at-a-time approach to limiting PFAS in drinking water.

#### Unique and shared challenges.

PFAS pose numerous challenges for drinking water providers and regulators. With an estimated 14,700 compounds classified as PFAS [7], the full extent of PFAS in water is likely underestimated by current analytical methods, which typically target only 20–30 compounds. The fifth cycle of UCMR (UCMR 5) will include 29 PFAS, including a range of both legacy and newer alternative PFAS [169]. Methods to estimate total PFAS (e.g., extractable organofluorine) or that target certain precursor compounds (e.g., total oxidizable precursor assay) are not widely applied to drinking water. Although thousands of water systems have discovered PFAS contamination, the full extent is unknown as testing has been inconsistent [160].

The cost of PFAS monitoring and treatment itself places substantial financial burdens on PWSs, especially those serving small and low-income communities. For example, the Hyannis Water System in Barnstable, MA, which serves 14,000 customers and includes environmental justice neighborhoods, has spent over $20 million to install granular activated carbon (GAC) treatment on 11 groundwater wells and will incur annual operating costs of $800,000 [170]. The cost for some large water systems could exceed $1 billion over time [171]. The most common PFAS treatment methods used by PWSs (GAC, ion exchange, reverse osmosis) are non-destructive, creating substantial quantities of contaminated filter media and wastes. High-temperature incineration under carefully-controlled monitoring and conditions has been reported in limited studies to largely break down PFAS and regenerate GAC filter media for reuse [172]. However, additional in-depth study is needed to confirm the efficacy of incineration/regeneration at fully destroying PFAS in spent GAC media under a variety of conditions, and use of such incineration has raised concerns about the sustainability of long-distance transport of spent GAC and air emissions from incineration potentially exacerbating exposures in environmental justice communities. New destruction methods such as super critical water oxidation are currently not commercially available, but in the future may provide a path forward to treat contaminated media and waste [173].

Many scientists, regulators, and advocates support class-based approaches to restricting PFAS [174]. Others, including industry representatives, have argued that applying a class-based approach to PFAS in drinking water is complicated by a lack of toxicity data for many PFAS and the different potencies of individual PFAS compounds. Shared challenges also include the lack of health assessments and occurrence data for the enormous class of PFAS that continues to grow literally daily. Finally, addressing PFAS contamination will require a concerted effort to limit PFAS manufacturing to avoid new sources of PFAS and new regulatory approaches that assess individual and combinations of PFAS, enhanced and more widespread testing to understand the true scope of water contamination, and development of new remediation methods to degrade PFAS without creating new exposure risks.

### Uranium

#### Sources and health effects.

Uranium (U) occurs naturally in the earth’s crust, with water contamination resulting from geochemical processes. Exposure to uranium in the US population is widespread; 74% of NHANES participants from 2001 to 2010 had detectable concentrations of U in their urine. Urine is the preferred biomarker to assess chronic exposure in populations with constant exposures, as previous studies have identified a good correlation between urine U and environmental U in water, air and food [175–178]. Drinking water remains the main route of U exposure in the US [179].

In the human body, uranium is rapidly distributed and accumulates in the bone and the kidneys, which are the main target organs that have been used in determining chemical toxicity for water standards [180]. Alpha radiation from uranium decay is classified as carcinogenic, and increasing epidemiological evidence shows that exposure to uranium in its metallic form is associated with chronic kidney disease, as well as neurologic, reproductive and cardiovascular toxicity [181–183]. While most epidemiologic studies have been conducted in occupational populations with high levels of exposure [184, 185], recent work on community level exposures in Indigenous communities exposed to uranium mine waste has identified more sensitive endpoints including cardiovascular disease and immune dysfunction [186, 187], and is supported by laboratory and animal uranium exposure model studies [188, 189]. Prior development of drinking water standards incorporated the long-held assumption that ingested uranium is very poorly absorbed from the gut. However, more recent studies suggest the higher resulting exposure of regulatory immune cells lining the gut may still result in central dysregulation of the immune system [190]. Thus, additional epidemiological studies are needed to better understand and characterize the adverse health effects of uranium drinking water exposure at the moderate and low exposure levels common in the general population, as well as to identify vulnerable subpopulations.

#### Exposure risk profile.

Uranium mobilization is influenced by the redox environment and increases in oxic groundwaters and in the presence of carbonate complexes, which can lead to the persistence of uranium in drinking water even after treatment to remove chemical contaminants [191, 192]. U is consistently found co-occurring with other metals in groundwater, mostly arsenic and selenium [193–196]. Anthropogenic activities, including mining of uranium ore, producing phosphate fertilizers, and military operations, can lead to increased uranium contamination in drinking water [177, 181, 197]. Depleted uranium (DU), a radioactive byproduct obtained from uranium enrichment and primarily derived from human activities, is one of the contributors to increased U exposure through drinking contaminated water and inhalation of DU aerosols. DU is used for the production of military and hospital equipment and has a half-life of millions of years [177]. Exposure to DU has been associated with renal, neurological and adverse developmental effects in previous observational studies [198, 199]. As a result, U concentrations in water supplies and air are highest in regions with natural geogenic uranium presence and redox conditions that facilitate its release, as well as intense mining, industrial, and military activities.

#### US regulatory context.

U is widely detected in private domestic wells and CWSs across the US. According to data from the US National Water Information System (NWIS), 50% of domestic wells in the US have detectable concentrations of U, with ~4% of wells exceeding the EPA MCL of 30 μg/L [200]. Uranium is also detected in 63.1% of regulated CWSs, with ~2% of CWSs exceeding the MCL [193]. Nationwide, CWSs reliant on groundwater have higher mean U concentrations (4.67 μg/L), compared to those reliant on surface water (1.79 μg/L) [193]. Of the 161,000 abandoned hard rock mines in the Western US states, U was the second highest prevalence of primary ore mined, creating the expectation of regionally higher concentrations of uranium in those regions, regardless of surface or groundwater sources, resulting from both anthropogenic contamination and natural mineralization [20].

#### Unique and shared challenges.

Several sociodemographic and geographical inequalities in U concentrations at the CWS level have been documented. Nationwide, the mean concentration of uranium in 2000–2011 was 4.4 μg/L [193]. Higher mean concentrations of uranium were detected in CWSs serving populations less than 500 (5.04 μg/L), those serving communities characterized as “Semi-Urban Hispanic” (10.04 μg/L), and CWSs serving communities in the Central Midwest (8.04 μg/L) and Southwest (9.13 μg/L) regions [193]. At the county level, a recent nationwide study identified that higher proportions of residents who self-identify as Hispanic/Latino, American Indian and Alaskan Native were associated with higher uranium in CWSs, after adjustment for income and education [201]. Previous studies in the Navajo Nation have documented higher concentrations of uranium and other toxic metals in drinking water sources compared to the rest of the US [194–196]. At least 12% of unregulated water sources in the Navajo Nation have uranium levels above the MCL of 30 μg/L [202]. Consistent with the exposure data, epidemiological studies have identified 2–3-fold higher urinary levels of uranium among Navajo people compared to the general population levels documented in NHANES [203], and urinary uranium concentration among pregnant women living on Navajo Nation in the Southwest are 2.67 to 2.80 times higher compared to the general US population [156]. Exposures related to private wells are a key challenge.

## CROSS-CUTTING EXPOSURE ISSUES

The array of exposure risk profiles for these seven different but widespread contaminants reveals a constellation of common elements, listed in Table 4 and discussed below.

### Aging, deteriorating water infrastructure

Much US water infrastructure was first installed in the late Victorian period when the influx of workers to cities demanded an enormous housing boom. It is now over 100 years old, and some is closer to 150 years old. Even much of the infrastructure installed later into the 20^th^ century is now past its design life. The American Society of Civil Engineers gave America’s drinking water infrastructure a “C-” grade, highlighting a water main break every two minutes and an estimated 6 billion gallons of treated water lost each day [204]. Pipe breaks and leaks also reduce water pressure potentially causing back-siphoning of bacteria and other contaminants into the system [205]. Unlined cast iron especially can be plagued by biofilms that can harbor pathogens if not carefully maintained [205, 206]. Additionally, EPA recently estimated that there are 9.2 million lead service lines nationally [92]. The lack of funding and prioritization for replacing these lead pipes has resulted in a slow pace of replacement posing public health risks particularly to vulnerable populations such as inner-city children [115, 207, 208].

Most US drinking water treatment plants provide conventional treatment including coagulation, sedimentation, sand filtration, and chlorination. EPA’s most recent Community Water System Survey found that less than 10% of drinking water treatment plants use modern technologies such as ion exchange, granular activated carbon (GAC), ozone, UV disinfection, or membranes [209, 210]. Half of the groundwater-supplied water treatment facilities provide no treatment other than disinfection [209, 210].

Deteriorated and outdated infrastructure can present health risks because it can introduce contaminants into the water (e.g., with lead service lines or DBPs), the impaired integrity of distribution system pipes can allow for contamination and recontamination. Outdated or poorly maintained treatment also may be inadequate to meet the challenges of contaminated source waters and may introduce contaminants into finished waters. This article documents that millions of US residents consume drinking water containing chemical contaminants that often may pose significant health risks ranging from cancer to neurological disease and other sequelae. In addition, while we have not examined microbiological risks from drinking water in detail, the US Centers for Disease Control and Prevention (CDC) and others have estimated that 4–32 million cases of gastrointestinal illness each year are waterborne [211–213], associated with emergency department visits, hospitalizations, and deaths, and incurring billions of dollars in direct healthcare costs; the precise contribution of drinking water to these illnesses is sometimes difficult to confirm [214]. Aging infrastructure is a major risk for microbial contamination due to broken and leaking pipes, poor water pressure, uncontrolled biofilms, etc.

Fixing these challenges will be expensive. EPA’s most recent assessment, published in 2023, estimated that $625 billion is needed to maintain and improve the nation’s drinking water infrastructure over the next 20 years [92]. This may be a substantial underestimate; the American Water Works Association estimated that repairing, updating and replacing crumbling drinking water infrastructure will cost at least $1 trillion over the next 25 years [215].

As Table 1 shows, 91% of CWSs serve under 10,000 people. Special challenges arise for small and rural water systems, and particularly those serving disadvantaged communities, as they have limited resources and often lack the technically-trained staff and the economies of scale to address system problems. The condition of water infrastructure relates largely to social determinants of health.

### Children, schools and childcare settings

Children, particularly those who are very young, are especially vulnerable to many contaminants commonly found in drinking water such as lead, arsenic, and nitrates [216]. Contamination can occur due to source water contamination, water delivery infrastructure or plumbing components containing lead, and/or inadequate water treatment, testing, and remediation practices. Some exposures can have severe and long-lasting health consequences [216]. Nationally, 1.5–2% of households are likely to have elevated concentrations of metal(loids), including lead, arsenic, and copper, in their drinking water [217].

In 2019, more than half of US children aged 5 or under (59%) not enrolled in kindergarten were in a non-parental care arrangement, where they are likely to consume water or food prepared on-site. Most of these children were cared for in a day-care, preschool, or similar facility (62%) or received care in a private home (20%) [218]. Additionally, nearly 50 million students attend school in the US, of which, in 2021, the majority (49.5 million) were in public school systems [219]. Schools that have their own water systems are regulated under Federal laws [36]. A GAO report found that 18% of reported exceedances of the Action Level occurred in schools and day care centers with their own water supplies; GAO considers this an underestimate [111].

However, most schools (89%) obtain their drinking water from a CWS [220]. Oversight and testing for contaminants in drinking water in educational settings like schools or childcare settings have historically been left to the states [218, 221]. Lead concentrations are strongly related to how long the water has stagnated, so schools and childcare facilities -- where the water can sit in pipes for 12 or more hours overnight and longer on weekends, holidays, and vacations – can present high potential exposure risks. Data collected in schools in US states and the District of Columbia have shown detectable levels of lead in school drinking water, including several exceedances of the Action Level of 15 μg/l [222–224]. Testing in 4005 childcare facilities in North Carolina found at least 1 tap water source exceeded 1 μg/l and 10 μg/l at 56% and 12% of facilities, respectively [225]. Reliance on well water may also be a risk factor for elevated lead concentrations in some educational facilities [225].

Several national efforts are underway to address lead in drinking water in educational settings. In 2016, as part of the Water Infrastructure Improvements for the Nation (WIIN) Act, a program was authorized to make funding available to states, territories, and Tribes to assist local agencies in voluntary testing for lead contamination in drinking water at schools and childcare facilities. This program was expanded via the Bipartisan Infrastructure Law in 2021, to make funding available for installation of filters or other remediation actions in response to testing. In addition, EPA’s Office of Ground Water and Drinking Water has developed resources to assist state programs and individual schools and childcare facilities in their efforts to reduce lead in drinking water. As of January 2023, the EPA reports testing in more than 12,500 educational facilities serving more than 3.5 million persons, enabling needed remediation to ensure lead-safe drinking water in educational settings [226].

As part of EPA’s recent Lead and Copper Rule Revisions (LCRR), over a 5-year period, CWSs must conduct sampling at 20% of elementary schools and 20% of childcare facilities per year and conduct sampling at secondary schools on request for 1 testing cycle [99]. For elementary schools, CWSs must test 5 outlets per school; CWSs must only test 2 outlets at childcare centers. Any follow up testing after the first test after 5 years is only upon request from the school. There is no mandatory notification of results to the parents, teachers, or children, and what actions, if any, the school system or CWS will undertake is unclear.

### Climate change

Climate change is likely to increase the occurrence of intense droughts, water scarcity, severe fires, rising sea levels, flooding, melting polar ice, and catastrophic storms [227]. These will have direct and indirect effects on the provision and quality of drinking water. Droughts will increase demand and reduce supply for water. Wildfires require water for control and can also pollute water sources. Higher temperatures will make higher water efficiency increasingly important to public water systems [228]. Rising sea levels increase intrusion of salt water into coastal aquifers and can contaminate near-coastal surface and ground water supplies. Chemical pollutants and pesticides that became airborne and deposited in glaciers, including banned persistent organic pollutants such as polychlorinated biphenyls; melting glaciers are now discharging the chemicals back into the surroundings and water bodies [229]. Global warming increases water temperatures and thus water lead levels, at the same time that higher temperatures will increase water consumption [121]. The acidification (lowered pH) anticipated for all environmental media will increase lead mobility and bioavailability.

### Disparities in access to clean, reliable, safe drinking water

Racial and ethnic disparities in some drinking water contaminant exposures, such as arsenic, uranium, lead and nitrate, are widely documented at the national, regional, or local levels in the US. These disparities mimic the inequities evident in housing, education, employment, earnings, health care, criminal justice, and environmental burdens and are likely underlined by structural racism [230, 231]. These inequities impact many health outcomes through sustained exposures to toxic environmental assaults, including drinking water contaminants, socioeconomic factors, and psychosocial stressors [232, 233]. The term ‘structural racism’ refers to the “totality of ways in which societies foster racial discrimination, through mutually reinforcing inequitable systems (in housing, education, employment, earnings, benefits, credit, media, health care, criminal justice, and so on).” [231, 234] Structural racism operates through a complex, multilevel, inter-active mechanism driven by factors across the natural, built and sociopolitical environment [235, 236]. While common patterns are evident for several particular drinking water contaminants and for overall drinking water system violation rates, the specific mechanisms producing these disparities vary across geographic regions and drinking water contaminants, rural versus urban environments, as well as other aspects of communities’ sociodemographic make-up [237, 238].

Major drivers of disparities in drinking water contaminants include selective enforcement of drinking water regulations, the exclusion of minorities in unincorporated areas from municipal boundaries and regulated water services (‘underbounding’ of communities of color), the direct withholding of resources and infrastructure investments, and the linguistic isolation of communities [116, 130, 235]. General childhood lead exposure, to which drinking water is now a major contributor, demonstrates the effect of structural racism in creating and reinforcing health disparities [18, 239]. The well-documented and publicized events in Flint, Michigan, resulted from intentional changes in water treatment and water supply sources, selective infrastructure investments and inappropriate tap sampling for compliance monitoring [15, 102].

Nationwide studies have also identified county-level racial and ethnic composition as a proxy for higher concentrations of other toxic chemicals in public drinking water [201, 240]. After considering socioeconomic, educational, and social vulnerability factors, communities with a higher proportion of non-Hispanic Black, Hispanic/Latino, Indigenous, and other minoritized racial and ethnic population groups, are exposed to higher levels of arsenic and uranium in their drinking water, two toxic metals with no known safe level of exposure [193, 201]. Similarly, nitrate concentrations are highest in CWSs serving communities with higher proportions of Hispanic/Latino residents [129], and PFAS were more frequently detected in CWSs serving higher proportions of Hispanic/Latinx and non-Hispanic Black residents [163]. Mounting evidence supports that current US public drinking water infrastructure, management, and regulatory action does not adequately protect communities of color from elevated contaminant exposures across a wide range of contaminants [116, 193, 201, 241].

### Inadequate health assessments

The US government uses risk assessments to determine priorities among competing needs, including health and environmental requirements. Outdated or inadequate health assessments bias governmental decisions and generate spurious results causing poor choices in determining priorities. The inadequacy of the assessments of drinking water contaminants discussed in this article include both outdated health data and the huge number of substances that have no health assessments at all. For widespread drinking water contaminants such as nitrate, lead and DBPs, the outdated health evaluations reflected in the drinking water standards mean that millions of Americans may have unsafe exposures, including in CWSs complying with current EPA standards. For classes of drinking water contaminants such as most DBPs, fracking-related substances and PFAS, identification and characterization of all of the chemical constituents has still not even occurred.

### Large number of substances

DBPs, fracking-related substances and PFAS constitute categories of separate chemicals predicted to number in excess of 15,000. Neither the full identity nor characterization of these substances are currently known, so health data are largely unknown also. However, in each category, sufficient toxicity and epidemiological data are available to suggest enormous potential health risks for the millions of exposed Americans. In addition, many of these substances are difficult and/or expensive to monitor for raising the added specter of financial burden. Of course, these will bear disproportionate burdens on low-income and disadvantaged communities.

### Preponderance of small water systems

Small water systems are generally defined as those serving 10,000 or fewer customers [242]. More than 90% of US CWSs are small. (Table 1) Many small systems face financial and operational challenges in providing drinking water that meets EPA standards [242]. Particular risks or complications for small systems include geographic dispersion and long distances, limited operational and technical resources, inadequate treatment capacity, affordability constraints), strained management demands, and communication needs [243]. The SDWA authorizes the potential use of Small System Variances to address small system challenges; these may, of course, constitute local exposure risks. Specifically, in 2002, Congress required EPA to re-evaluate EPA’s Small System Variances policy due to the high cost of arsenic treatment in small communities; in response, EPA may alter its affordability guidelines [243]. In addition, small systems may be at increased vulnerability to a variety of attacks, including contamination with deadly agents; physical attacks, such as the release of toxic gaseous chemicals; and cyberattacks [244]. Small systems face increased risks related to nitrate, DBPs, fracking and arsenic contamination. Furthermore, climate change will impose a larger burden on small and financially constrained water systems related to economies of scale and constrained resources.

### Uneven enforcement of US drinking water standards

EPA has established national primary drinking water standards for about 100 contaminants [36]; many were set under specific Congressional mandates [36, 245]. After EPA establishes an MCL, states can obtain “Primacy” to implement and enforce them, upon approval by EPA [245]. Each state and recognized Tribal government may apply to EPA for Primacy, formally known as Primary Enforcement Authority, by showing that it has adopted drinking water standards as stringent as the EPA standards and has the authority and capacity to implement and enforce those standards. Once a Primacy agency has received EPA approval, it has the primary responsibility for administrating and enforcing regulations. EPA retains oversight authority to ensure that Primacy agencies are complying with federal rules, and EPA also can file an enforcement action if a Primacy agency has failed to do so. Forty-nine states have Primacy for drinking water, although EPA continues to have Primacy in Wyoming, the District of Columbia, and in Indian country with the exception of the Navajo Nation (the only Tribe with Primacy) [246].

Enforcement of federal drinking water standards is inconsistent across the US [116, 117]. and violations of the EPA drinking water standards are frequent. EPA documents over 40,000 violations annually for US drinking water systems, of which about 2600 PWSs faced formal enforcement actions (such as an administrative order) in 2022 for current and past violations. More than 27,000 violators reportedly received “informal” enforcement such as a reminder or warning letter [247]. These likely underestimate actual violations. An EPA audit published in 2008, for example, showed that only 8% of treatment technique violations of the LCR contained in state files were reported to the EPA [248]. A 2011 Government Accountability Office review confirmed widespread under-reporting of drinking water violations, with only 16–72% of violations reported [113]. The Natural Resources Defense Council (NRDC) reviewed EPA-reported violations and determined that in 2015 alone, there were more than 80,000 reported violations of the SDWA by >18,000 CWSs serving nearly 77 million people [117]. Another NRDC survey found that that in 2018, 2019 and 2020, 28 million people were served by 7595 CWSs with 12,892 lead violations [115].

Drinking water violations and inconsistent government response and resolution of violations disproportionately affect low-income communities and communities of color [116, 241]. A 2017 study found that in communities with higher populations of black and Hispanic individuals, drinking water health violations are more common, and that in the poorest of communities race and ethnicity matter most in determining drinking water quality [116]. A 2018 national study similarly found that areas with greater populations of low socio-economic status, minority populations, and uninsured populations were more likely to have initial and repeat drinking water violations [249]. Race, ethnicity and spoken language of the population have been identified as the strongest factors for drinking water violations and slow resolution of these violations [116].

### Water access and safety for US Indigenous communities

Indigenous communities in the US face significant challenges in accessing and ensuring the quality of their drinking water. Despite their status as sovereign nations under both the SDWA and the Clean Water Act (CWA), many Tribes continue to struggle with establishing water quality standards under the CWA to protect surface waters on Tribal lands from upstream or local water pollution discharges. This has implications for the health of Tribal populations, and absent Tribal adoption of CWA water quality standards for their surface waters, many Tribal governments lack authority to enforce those standards to protect the source waters used by water systems serving their populations.

In addition, primary authority to oversee drinking water systems can be secured by Tribes through the same process by which EPA approves Primacy to States. The resource and monetary challenges associated with obtaining Primacy on Tribal lands are compounded by aging infrastructure and the significant distances over which clean water needs to be delivered to serve remote Tribal populations. Currently, approximately 80 Tribes have been delegated authority to establish their own CWA water quality standards to protect their surface waters [250]. However, full Primacy under the SDWA has been granted to only one Tribe to date: the Navajo Nation has been granted Primacy to regulate the operation of 170 PWSs on their lands (a mix of Tribal and privately-owned systems).

**Fig. 1:**
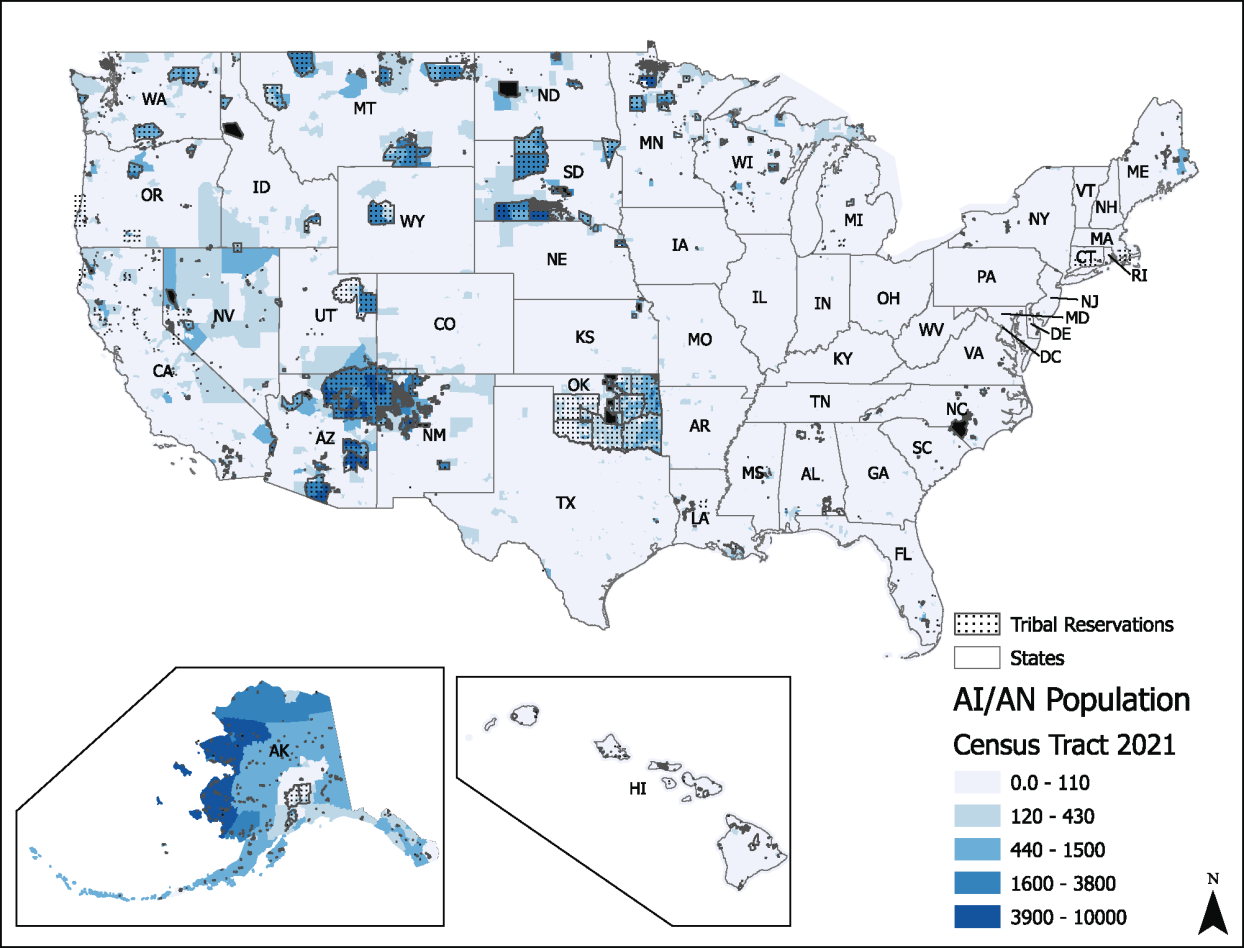
Tribal population within Tribal reservation boundaries in the US by census tract.

More than 1000 water systems serve over 1 million people in “Indian Country”, underscoring the lack of access to regulated water supplies for more than 50% of the Indigenous population. The estimated Indigenous population of the US, based on the 2020 Census, is 4.4 million of American Indian or Alaska Native lineage alone and 7 million including those with mixed race (of the former, 2.7 million live within Tribal reservations) (Fig. 1). Access to regulated drinking water in homes varies significantly across Tribes. For the Navajo Nation, the largest Indigenous population living on reservation (~170,000), up to 30% of reservation households lacked access to regulated water within their homes in 2022 [251].

Compliance and access with regard to Tribal water systems has improved from 2009 to 2021, but when comparing Tribal systems to the US as a whole, the magnitude of disparities has been virtually unchanged. In 2009, PWSs serving US Tribal populations had nearly double the percentage of reported significant health-based violations as did those in the US as a whole. Of those reported significant health-based violations in 2009 (~14% of Tribal systems vs. ~5% of all US systems), 40% of Tribal violations were chemical contaminants, compared to 20% of all US system violations [252]. In 2021, the overall rates of significant health-based violations have decreased for both groups, although Tribal systems continue to have approximately twice the percentages of systems with significant health-based violations (6% Tribal vs 3% all US, 2021) [253]. Furthermore, as discussed above, there is often substantial under-reporting of violations to EPA’s database.

The biggest difference relates to infrastructure disparities faced by Tribes. In 2021, for instance, many of the health-based violations in Tribal systems involved infrastructure and resource disparities, such as water sources in proximity to failing septic systems with pathogen contamination risk and other sanitary issues constituting violations of EPA’s Ground Water Rule; in the rest of the US, chemical contamination is more common. As Tribes assume increasing regulatory authority, access to clean sources and adequate water for Tribal systems remains challenging. As of January 2023, $580 million in the US has been authorized for 15 Tribes to settle water rights issues, and for pumping and water distribution [253]. Water access is challenged by the long distances to reach homes and both mineralization and the legacy of anthropogenic contamination by industries such as mining. In the Western US, home to more than 50% of the Indigenous population, we estimated that 600,000 Indigenous people live within 10 km of at least one of the 161,000 documented abandoned hard rock mines [20, 254]. EPA estimates these sources of mixtures of heavy metals have contaminated more than 40% of the surface waters in the Western US. Without access to regulated PWS or CWS water, the reliance on unregulated sources for drinking water traditionally has been on surface sources, private, or livestock wells. These unregulated sources also evidence impacts from mineralization and abandoned mines, creating clusters of sources exceeding MCLs for multiple heavy metals including arsenic and uranium, as well as barium, cesium, and other metals [202].

## CONCLUSIONS

While the US and many other countries have greatly reduced acute risks from drinking water microbial contamination, chemical contamination of drinking water is associated with a wide range of chronic adverse health impacts including cancer and developmental, neurological and reproductive effects. A full understanding of pollutant-specific exposures and risks is hindered by limited availability of data on the occurrence, concentrations, and toxicity of these diverse legacy and emerging chemical contaminants. Further, there are many complex factors that influence the exposure and risk profiles of chemical contaminants in drinking water, including well depth, hydrogeological factors, types of distribution systems and disinfection treatments, and socioeconomic and demographic characteristics of impacted populations. The seven contaminants and contaminant groups presented here represent a tiny fraction of the thousands of regulated and unregulated chemical agents present in drinking water. This review illustrates the complexities of the array of chemical hazards in our drinking water and highlights the need for a concerted effort to invest in upgrading our drinking water infrastructure, strengthen drinking water standards, develop and implement enhanced water treatment, collect and disseminate monitoring data, and require more stringent chemical safety testing to support the welfare of all US residents. Furthermore, our analysis underscores that a focus on source water protection would be more effective than the post-hoc treatment that is now necessary to protect public health.

## Supplementary Material

Reporting Checklist

## Figures and Tables

**Table 1.** US public water systems (PWSs) by system size and population served, quarter 4, 2022^a^.

	System size	Number of systems (%)	Population served (millions) (%)
Community water systems^b^	Very small to medium^c^	45,202 (91%)	52 (16%)
	Large^d^	4030 (8%)	116 (36%)
	Very large^e^	455 (1%)	151 (47%)
Noncommunity water systems^f^	Very small to medium^c^	101,829 (99.9%)	16.4 (84%)
	Large^d^	65 (0%)	1.5 (8%)
	Very large^e^	5 (--%)	1.6 (8%)
Total all PWSs	Very small to medium^c^	147,031	68
	Large^d^	4095	118
	Very large^e^	460	153
		151,586	339

a Based on data from the Government Performance and Results Act (CPRA) tool viewable at https://www.epa.gov/ground-water-and-drinking-water/drinking-water-performance-and-results-report.

b Community Water System: serves the same 15 or more service connections or an average of at least 25 or more people for at least 60 days a year.

c Serving 10,000 customers or fewer.

d Serving 10,001–100,000 customers or fewer.

e Serving over 100,000 customers.

f Non-Community Water System: serves at least 15 service connections or an average of at least 25 people for at least 60 days a year, but the population changes, such as campgrounds, restaurants, and schools.

**Table 2.** US public water systems (PWSs) by water source quarter 4, 2022^a^.

	Source water type	Number of systems	Population served (millions)
Community water systems^b^	Groundwater	37,936	91
	Surface water	11,714	229
Noncommunity water systems^c^	Groundwater	98,689	17.3
	Surface water	3038	2.2
Total all PWSs	Groundwater	136,625	108
	Surface water	14,752	231
	All sources	151,377	339

a EPA. Safe Drinking Water Information System, GPRA [Government Performance and Responsibility Act] Inventory Report, 2022 Q4 [Quarter 4], available online at https://obipublic.epa.gov/analytics/saw.dll?PortalPages&PortalPath=/shared/SFDW/_portal/Public (visited February 22, 2023). Note that due to rounding, the total percentages may not add up to precisely 100%.

b Community Water System: serves the same 15 or more service connections or an average of at least 25 or more people for at least 60 days a year.

c Noncommunity Water System: serves at least 15 service connections or an average of at least 25 people for at least 60 days a year, but the population changes. Examples: campgrounds, restaurants, etc.

**Table 3.** EPA regulations of drinking water contaminants.

Contaminant group	Substance	MCL,^a^ MRDL^b^ or TT^c^	Standard (mg/l)
Disinfectants	Chloramines (as Cl2)	MRDL	4.0
	Chlorine	MRDL	4.0
	Chlorine dioxide	MRDL	0.8
Disinfection by-products	Bromate	MCL	0.010
	Chlorite	MCL	1.0
	Haloacetic acids	MCL	0.060
	Total Trihalomethanes	MCL	0.080
Inorganic chemicals	Antimony	MCL	0.006
	Arsenic	MCL	0.010
	Asbestos	MCL	(fibers >10 micrometers) 7 million fibers per liter (MFL)
	Barium	MCL	2
	Beryllium	MCL	0.004
	Cadmium	MCL	0.005
	Chromium	MCL	0.1
	Copper	TT	^d^; Action Level = 1.3
	Cyanide	MCL	0.2
	Fluoride	MCL	4.0
	Lead	TT	^e^
	Mercury (inorganic)	MCL	0.002
	Nitrate	MCL	10
	Nitrite	MCL	1
	Selenium	MCL	0.05
	Thallium	MCL	0.002
Microorganisms	Cryptosporidium	TT	^f^
	Fecal coliform & E. coli	MCL	^g^
	Giardia lamblia	TT	^h^
	Heterotrophic plate count	TT	^i^
	Legionella	TT	^j^
	Total Coliforms	MCL	5.0%^k^
	Turbidity	TT	^l^
	Viruses	TT	^m^
Organic chemicals	Acrylamide	TT	^n^
	Alachlor	MCL	0.002
	Atrazine	MCL	0.003
	Benzene	MCL	0.005
	Benzo(a)pyrene (PAHs)	MCL	0.0002
	Carbofuran	MCL	0.04
	Carbon tetrachloride	MCL	0.005
	Chlordane	MCL	0.002
	Chlorobenzene	MCL	0.1
	2,4-D	MCL	0.07
	Dalapon	MCL	0.2
	1,2-Dibromo-3- chloropropane (DBCP)	MCL	0.0002
	o-Dichlorobenzene	MCL	0.6
	p-Dichlorobenzene	MCL	0.075
	1,2-Dichloroethane	MCL	0.005
	1,1-Dichloroethylene	MCL	0.007
	cis-1,2- Dichloroethylene	MCL	0.07
	trans-1,2, Dichloroethylene	MCL	0.1
	Dichloromethane	MCL	0.005
	1,2-Dichloropropane	MCL	0.005
	Di(2-ethylhexyl) adipate	MCL	0.4
	Di(2-ethylhexyl) phthalate	MCL	0.006
	Dinoseb	MCL	0.007
	Dioxin (2,3,7,8-TCDD)	MCL	0.00000003
	Diquat	MCL	0.02
	Endothall	MCL	0.1
	Endrin	MCL	0.002
	Epichlorohydrin	TT	^o^
	Ethylbenzene	MCL	0.7
	Ethylene dibromide	MCL	0.00005
	Glyphosate	MCL	0.7
	Heptachlor	MCL	0.0004
	Heptachlor epoxide	MCL	0.0002
	Hexachlorobenzene	MCL	0.001
	Hexachlorocyclopentadiene	MCL	0.05
	Lindane	TT	0.0002
	Methoxychlor	MCL	0.04
	Oxamyl (Vydate)	MCL	0.2
	Pentachlorophenol	MCL	0.001
	Picloram	MCL	0.5
	Polychlorinated biphenyls (PCBs)	MCL	0.0005
	Simazine	MCL	0.004
	Styrene	MCL	0.1
	Tetrachloroethylene	MCL	0.005
	Toluene	MCL	1
	Toxaphene	MCL	0.003
	2,4,5-TP (Silvex)	MCL	0.05
	1,2,4- Trichlorobenzene	MCL	0.07
	1,1,1- Trichloroethane	MCL	0.2
	1,1,2- Trichloroethane	MCL	0.005
	Trichloroethylene	MCL	0.005
	Vinyl chloride	MCL	0.002
	Xylenes (total)	MCL	10
Radionuclides	Alpha/photon emitters	MCL	15 picocuries per Liter (pCi/L)
	Beta photon emitters	MCL	4 millirems per year
	Radium 226 and Radium 228 (combined)	MCL	5 pCi/l
	Uranium	MCL	30 μg/l

a MCL: Maximum Contaminant Level. The highest level of a contaminant that is allowed in drinking water. MCLs are set as close to Maximum Contaminant Level Goals (MCLGs) as feasible using the best available treatment technology and taking cost into consideration. MCLs are enforceable standards.

b MRDL: Maximum Residual Disinfectant Level. The highest level of a disinfectant allowed in drinking water.

c TT: Treatment Technique. A required process intended to reduce the level of a contaminant in drinking water.

d TT for copper requires systems to control the corrosiveness of their water. If more than 10 percent of tap water samples exceed the action level of 1.3 mg/L, water systems must take additional steps.

e TT for lead requires systems to control the corrosiveness of their water. If more than 10 percent of tap water samples exceed the action level of 0.015 mg/L, water systems must take additional steps.

f 99% removal for systems that filter. Unfiltered systems are required to include Cryptosporidium in their existing watershed control provisions.

g A routine sample that is fecal coliform-positive or E. coli-positive triggers repeat samples. If any repeat sample is total coliform-positive, the system has an acute MCL violation. A routine sample that is total coliform-positive and fecal coliform-negative or E. coli negative triggers repeat samples.

h 99.9 percent removal/inactivation.

i No more than 500 bacterial colonies per milliliter.

j No limit.

k No more than 5.0 percent samples total coliform-positive in a month.

l For systems that use conventional or direct filtration, at no time can turbidity exceed 1 nephelometric turbidity unit (NTU), and samples for turbidity must be less than or equal to 0.3 NTU in at least 95 percent of the samples in any month. Systems that use filtration other than the conventional or direct filtration must follow state limits, which must include turbidity at no time exceeding 5 NTU.

m 99.9 percent removal/inactivation.

n Each water system must certify annually, in writing, to the state (using third-party or manufacturers certification) that when it uses acrylamide and/or epichlorohydrin to treat water, the combination (or product) of dose and monomer level does not exceed the levels specified, as follows: Acrylamide = 0.05 percent dosed at 1 mg/L (or equivalent); Epichlorohydrin = 0.01 percent dosed at 20 mg/L (or equivalent).

o Each water system must certify annually that when it uses acrylamide and/or epichlorohydrin to treat water, the combination (or product) of dose and monomer level does not exceed the levels specified, as follows: Acrylamide = 0.05 percent dosed at 1 mg/L (or equivalent); Epichlorohydrin = 0.01 percent dosed at 20 mg/L (or equivalent).

**Table 4.** Cross-cutting issues affecting seven contaminants of drinking water supplies.

Cross-cutting issue	Issue description	Evidence that contaminant impacted^a^						
		Arsenic	Disinfection byproducts	Fracking Chemicals	Lead	Nitrate	PFAS	Uranium
Aging infrastructure	Broken or leaking pipes, poor water pressure, leaded pipes		X		X			
Children, childcare facilities	Contaminants known to be present in schools and childcare settings; vulnerability of children to toxic effects				X	X		
Climate change	Droughts increase demand and reduce supply; wildfires and flooding contaminate source waters; higher temperatures and environmental acidification mobilize pollutants from environmental media	X	X		X			
Disparities	Distributive injustices related to inequities in exposure based on income, race/ethnicity, or other factors	X			X	X	X	X
Inadequate health assessments	Based on outdated data or lacking completely	X	X	X		X	X	X
Many small systems with contamination	Limited operational and technical resources, inadequate treatment capacity	X	X	X		X	X	
Numerous substances	Full identity or full toxicological characterization unavailable		X	X			X	
Indigenous populations	Issues of sovereignty and distributive injustices of pollutant sources	X				X		X
Uneven enforcement	Procedural justice issues related to violations	X			X	X		X
Inadequate ccurrence data	Lack of monitoring data		X	X	X		X	
Private well contamination	Chemicals known to contaminate private wells, which are unregulated	X		X	X	X	X	X

a An X indicates the availability of documented evidence of an effect on contaminant levels in water supplies. There may be additional impacts that are not yet well-documented.
