# Supplementary material for: US drinking water quality: exposure risk profiles for seven legacy and emerging contaminants
Source: J Expo Sci Environ Epidemiol. Author manuscript; Available in PMC 2024 Mar 8. (PMC10907308; doi:10.1038/s41370-023-00597-z)
Supplement: Reporting Checklist [file NIHMS1958690-supplement-Reporting_Checklist.docx]

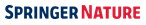


Corresponding Author name: _____Ronnie Levin________

Manuscript Number: ______  JESEE-23-4212.R1_________

**Reporting Checklist**

This checklist is used to ensure the quality, transparency, and reproducibility of published results. We require authors attest that these components have been considered and addressed.

| **Exposure Assessment Guiding Principle** | **Yes/No/Not Applicable** |
| --- | --- |
| Has the method to estimate exposure been described clearly? | Yes, varies by contaminant and described individually |
| Has the exposure assessment method been validated/evaluated as a proxy for exposure and is its validity or agreement with other methods described? | Yes, varies by contaminant and described individually |
| Is the time period over which the exposure assessment method is considered to be a proxy for exposure appropriate for the research question? | Yes, varies by contaminant and described individually |
| If exposure is modeled or measured, were all critical potential routes and sources of exposure considered? | Yes, varies by contaminant and described individually |
| If exposure is modeled, how does it vary over space and time and are necessary historical data incorporated? | Yes, varies by contaminant and described individually |
| If biomarkers are used as indicators of exposure, could the biomarker measurement have been affected by the outcome (i.e., reverse causality)? | N/A |
| Are the strengths and weaknesses of the exposure approach detailed and discussed? | Yes, varies by contaminant and described individually |
